# Extracellular vesicles as carriers for noncoding RNA-based regulation of macrophage/microglia polarization: an emerging candidate regulator for lung and traumatic brain injuries

**DOI:** 10.3389/fimmu.2024.1343364

**Published:** 2024-03-15

**Authors:** Zhihong Chen, Jingang Zhang, Yongli Pan, Zhongnan Hao, Shuang Li

**Affiliations:** ^1^ Department of Respiratory Medicine, The Third People’s Hospital of Longgang District, Shenzhen, China; ^2^ Department of Orthopedic, The Third People’s Hospital of Longgang District, Shenzhen, China; ^3^ Department of Neurology, Shandong Provincial Hospital, Shandong University, Jinan, Shandong, China; ^4^ Department of Neurology, University Medical Center of Göttingen, Georg-August-University of Göttingen, Göttingen, Lower Saxony, Germany

**Keywords:** extracellular vesicles, noncoding RNA, macrophage/microglia polarization, lung brain injury, traumatic brain injury

## Abstract

Macrophage/microglia function as immune defense and homeostatic cells that originate from bone marrow progenitor cells. Macrophage/microglia activation is historically divided into proinflammatory M1 or anti-inflammatory M2 states based on intracellular dynamics and protein production. The polarization of macrophages/microglia involves a pivotal impact in modulating the development of inflammatory disorders, namely lung and traumatic brain injuries. Recent evidence indicates shared signaling pathways in lung and traumatic brain injuries, regulated through non-coding RNAs (ncRNAs) loaded into extracellular vesicles (EVs). This packaging protects ncRNAs from degradation. These vesicles are subcellular components released through a paracellular mechanism, constituting a group of nanoparticles that involve exosomes, microvesicles, and apoptotic bodies. EVs are characterized by a double-layered membrane and are abound with proteins, nucleic acids, and other bioactive compounds. ncRNAs are RNA molecules with functional roles, despite their absence of coding capacity. They actively participate in the regulation of mRNA expression and function through various mechanisms. Recent studies pointed out that selective packaging of ncRNAs into EVs plays a role in modulating distinct facets of macrophage/microglia polarization, under conditions of lung and traumatic brain injuries. This study will explore the latest findings regarding the role of EVs in the progression of lung and traumatic brain injuries, with a specific focus on the involvement of ncRNAs within these vesicles. The conclusion of this review will emphasize the clinical opportunities presented by EV-ncRNAs, underscoring their potential functions as both biomarkers and targets for therapeutic interventions.

## 1 Introduction

Both lung injury and traumatic brain injury trigger a systemic inflammatory response, marked by the release of pro-inflammatory cytokines, chemokines, and other mediators. This inflammation exacerbates damage in both organs. Moreover, traumatic brain injury often requires mechanical ventilation, potentially causing ventilator-induced lung injury due to the mechanical forces exerted during ventilation, which can intensify lung inflammation and damage. Lung injury may lead to the release of inflammatory mediators and damage-associated molecular patterns into the bloodstream, disrupting the blood-brain barrier and increasing the brain’s vulnerability to secondary insults after traumatic brain injury. Additionally, severe traumatic brain injury is linked to neurogenic pulmonary edema, a type of non-cardiogenic pulmonary edema characterized by increased permeability of pulmonary capillaries, worsening lung function and contributing to respiratory failure in affected patients.

An integral component of both innate and acquired immunity is the class of immune cells referred to as macrophages/microglia, originating from bone marrow progenitor cells (1). Within the cellular context implicated in injury, macrophages/microglia display plastic traits, experiencing a process known as polarization. This encompasses transitions between specific functional phenotypes (M1/M2) triggered by environmental impact. The polarization of macrophages/microglia serves various roles in tissue repair and the maintenance of homeostasis. Additionally, it has a significant impact on numerous circumstances like traumatic brain injury and lung injury. Dysregulation of macrophage/microglia polarization can lead to chronic inflammation and contribute to pathological conditions (2, 3). The pathophysiological mechanisms underlying macrophage/microglia activation in brain and lung injuries are very different. Following traumatic brain injury, damage ensues to the surrounding tissues and cells. This damage results in the release of intracellular components and signaling molecules. In response to this physiological stimulus, microglia become activated, engaging in the clearance and repair of the affected area (4, 5). Furthermore, traumatic brain injury initiates an inflammatory response, causing the release of inflammatory factors and cytokines. These signaling molecules have the potential to activate microglia (4, 5). During lung injury, macrophages are activated as they recognize pathogens or foreign substances, coupled with the release of intracellular components from damaged lung tissues and cells (6, 7).

Despite clear pathophysiological distinctions, there is a great deal of similarity between these two disorders’ gene responses to damage. These commonalities apply to non-coding RNAs (ncRNAs) as well as RNAs that encode proteins. Emerging data from recent times suggests that ncRNAs produced from extracellular vesicles (EVs) play a crucial role as regulators of cell-to-cell communication in a shared signaling pathway, have garnered noteworthy attention in regulating the polarization of macrophages/microglia in these two pathologies (8–10). EVs are nanosized vesicles enclosed by a double-layered membrane, released by virtually all cell types (11). These vesicles carry RNA, DNA, proteins, and lipids, serving as regulators of cell-to-cell communication by facilitating the transport of mRNAs and proteins between cells (12, 13). ncRNAs, a diverse class of RNAs, encompass various subclasses. The primary classification criterion for these molecules is their size (14). Notably, as integral components, ncRNAs are selectively concentrated within EVs (12), and the transferred ncRNAs in EVs have a vital part in regulating distinct facets of the initiation and advancement of brain and lung injuries. Lung and brain injuries represent critical areas where the role of EVs and ncRNAs has garnered significant attention in recent research. For instance, EVs and ncRNAs have been implicated in various pathophysiological processes associated with lung injury, such as inflammation, fibrosis, and tissue repair. Similarly, in the context of brain injuries, EVs and ncRNAs play crucial roles in neuroinflammation, neuroprotection, and neural regeneration. By focusing on these specific organ systems, the authors aim to provide a comprehensive understanding of the mechanisms underlying EV and ncRNA involvement in injury pathogenesis and identify potential therapeutic targets. In light of the effects of EV-ncRNAs on regulating macrophage/microglia polarization has been revealed in brain and lung injuries. This review aims to consolidate recent progress in the exploration of EV-ncRNAs spanning from traumatic brain injury to lung injury, with a specific focus on the modulation of macrophage/microglia polarization. Additionally, we delineate the potential clinical applications of EV-ncRNAs and their potential functions as both biomarkers and targets for therapeutic interventions.

## 2 Macrophage/microglia polarization in lung and traumatic brain injuries

### 2.1 Polarization *of macrophages* in acute lung damage

Macrophages, a prominent subset of myeloid cells, represent a significant group of innate immune cells. They exhibit considerable heterogeneity and phenotypic specialization while playing a critical role in innate immunity across diverse tissues (15). Originally, there was a prevalent belief that tissue macrophages originated from monocytes in circulation (16, 17). However, the revelation of localized macrophages in tissue in various bodily systems, stemming throughout the growth phase of the embryo, from the yolk sac, has fundamentally altered researchers’ perspectives on the origin of macrophages (18, 19). Macrophages can originate either by differentiating from monocytes in circulation that come from bone marrow stem cells or by deriving from primitive macrophages that originate from the maternal liver and yolk sac of the embryo (20). Based on their lung location, macrophages can be categorized into two types: alveolar macrophages, situated on the alveolar interior surface and directly exposed to the external surroundings, having a vital part in the initial immune response; and interstitial macrophages, commonly related to airways, nerves, and vessels (21, 22).

Under the influence of distinct tissue microenvironments, macrophages undergo polarization, giving rise to M1 and M2 subtypes with distinct functions. The balance between the M1 and M2 phenotypes dictates the outcome of an organ during inflammation or injury. M1 macrophages engage in antimicrobial properties, generate inflammatory-promoting factors, and mediate organ injury, while M2 macrophages generate factors that reduce inflammation and facilitate organ recovery (23). M1 macrophages, frequently described as “classically activated” (proinflammatory) macrophages,” become activated through stimuli like colony-stimulating factor for granulocyte-macrophage colonies and TLR ligands like LPS and Th1-type cytokines (20). M1 macrophages secrete an abundance of factors that promote inflammation, including TNF-α, IL-6, IL-1β, IL-12, IL-23, CXCL1-3, CXCL-5, and CXCL8-10 (24). Additionally, M1 macrophages exhibit high expression of CD40, CD68, CD80, and CD86 (25). On the other hand, Th2 cytokines like IL-4 and IL-13, along with anti-inflammatory cytokines like IL-10 and TGF-β, cause M2 macrophages to develop (26). These macrophages exhibit an anti-inflammatory character of factors defined by reduced IL-12 production and elevated levels of chemokine CCL18, Arg-1, IL-10, and TGF-β (27). An increasing amount of data indicates that discerning the polarized conditions of macrophages and causing the M1 to M2 transition, could serve as an innovative therapeutic approach for acute lung injury (8, 27).

### 2.2 Microglia polarization in traumatic brain injury

Particularized macrophages found only in the central nervous system are called microglia (28). Through fate mapping, it was discovered that microglia trace their origins back to cell progenitor of myelo-erythroids found in the yolk sac of mammals (29). These ancestors’ cells undergo migration into the brain during the period in animals, around embryonic days 9.5 and 14.5 (30, 31). Unlike macrophages within different tissues, the density of microglia continues constant throughout the lifespan of both mice and humans, sustained by the local proliferation of microglial cells (29). Microglia typically display a highly branched morphology during their inactive state, featuring numerous short and slender processes (28). These processes create an extensive contact area, reaching into the nearby environment, and positioning microglia strategically to sense and monitor local environmental changes. In reaction to a cranial trauma, microglia own the capacity to provoke swift and significant alterations in gene expressions, cell forms, and functional characteristics. These changes are collectively termed “activation of microglia” (28, 32). Upon activation, microglia demonstrate the ability to migrate toward lesions, engulf broken cells or tissue fragments through phagocytosis, coordinate inflammation of the nervous system by releasing crucial agents of inflammation, and secrete various substances, including oxygen species that are reactive and neurotrophic. These substances can have both advantageous and adverse consequences on the encircling the cells (33).

Based on the intracellular dynamics and protein production of microglia, activation of microglia was previously classified into classical proinflammatory M1 or alternative anti-inflammatory M2 states. Switching between M1 and M2 phenotypes holds practical significance, as fostering the M2 haplotype can aid in tissue restoration by reducing variables that cause inflammation. M1 activation is considered a proinflammatory and neurotoxic state, triggered by the induction of TLR and IFN-γ signaling pathways. Characteristic markers of the M1 phenotype include CD16, CD32, CD86, and the inducible forms of iNOS. Microglia of the M1 phenotype release inflammatory-promoting molecules, including TNF-α, IL-6, IL-12, IL-1β, and NO, thereby intensifying inflammation and contributing to tissue damage (34–36). In contrast, the alternative M2 microglia, identified by the manifestation of indicators like CD163, TGF-β1, IL-10, CD206, Arg-1, Ym-1, and FIZZ-1, contribute to the suppression of inflammation, clearance of debris, and facilitation of tissue remodeling (37–39). It’s worth mentioning that, according to the current understanding of M2-polarized microglia within the brainstem, in accordance with how cells function, the M2 subtype is additionally divided into M2a, M2b, or M2c subcategories. M2a microglia exhibit a robust association with IL-13 and IL-4, showcasing potent anti-inflammatory properties. Additionally, these cells generate substantial quantities of arginase-1, Ym-1, CD206, and Fizz1 (40), in contrast to the M2b phenotype, which lacks the ability to produce the latter (41, 42). The M2c-like subtype, representing obtained termination, plays a role in immune regulation, tissue repair, and remodeling (43).

## 3 The chemical makeup of ncRNAs and EVs

### 3.1 EV Segmentation and related features

EVs are membranous structures discharged into the extracellular environment, originating from either the endosomal system or the plasma membrane of various cell types (44). Research has found that such vesicles can be created and distributed by various types of cells like neurons, glial cells, immune cells, mesenchymal stem cells, and others, into serum, amniotic fluid, plasma, urine, saliva, and cerebrospinal fluid (45). Microvesicles, apoptotic bodies, and exosomes are the three primary forms of EVs based on their size and biological origin. Exosomes, with a size range of 30-150 nm, originate as fluid within multivesicular structures as intraluminal vesicles and are discharged following their union with the plasma membrane. Microvesicles (50-1000 nm) and apoptotic bodies (100-5000 nm) are both larger in size. They develop as a result of the plasma membrane dividing and budding outward, or blebbing after apoptosis, respectively (46). The main active components responsible for the biological effects of EVs are composed of proteins, lipids, miRNA, and other various constituents. After donor cells synthesize and release EVs, the active components in the EVs are delivered to the cytoplasm of target cells through indirect binding to signal receptors or direct merging with the membrane of the cell, thereby presiding over intercellular communication and exerting biological effects (47). EVs biogenesis and secretion can occur through either the Endosomal Sorting Complex Required for Transport (ESCRT)-dependent pathway (48, 49) or the ceramide-dependent pathway (50). The ESCRT family encompasses several members, including ESCRT-0, ESCRT-I, ESCRT-II, and ESCRT-III, as well as ESCRT-associated proteins like Alix (51). The ESCRT-dependent pathway is initiated upon the presence of ubiquitinylated membrane proteins in early endosomes. Subsequently, the ESCRT complexes collaborate to facilitate the sorting of ubiquitinylated proteins into EVs (52).

### 3.2 Classification and associated properties of ncRNAs

Human genome sequencing reveals that most of the genome is DNA that does not code for proteins, with just over 3% of the genes encoding proteins (53). ncRNA, which cannot produce proteins, is essential for controlling a number of processes that are both physiological and pathological (54–56). There are various types of ncRNAs, including long ncRNAs (lncRNAs), microRNAs (miRNAs), PIWI-interacting RNA (piRNA), small nucleolar RNA (snoRNA), small interfering RNAs (siRNA), and circular RNAs (circRNAs) (57). miRNA, which are 19-25 nucleotides in length, belongs to a class of small ncRNAs (58). The gene for miRNA is generated primary miRNA transcripts (pri-miRNA) by transcription by RNA polymerase II. The ribonuclease Drosha then cleaves pri-miRNA into Pre-miRNA, or roughly 70 nt stem-loop precursor miRNA. Subsequently, these precursor miRNAs are further processed by the cytoplasmic ribonuclease Dicer to generate mature miRNA. By matching complementary bases, miRNA can identify target mRNAs. They direct the silencing complex to either destroy the target mRNA or prevent its translation, depending on the degree of complementarity, thereby regulating mRNA stability and translation to mediate post-transcriptional gene expression (59). Typically, the 3’ untranslated region (3’UTR) sequence of its target mRNA is where miRNA attaches itself, leading to deterioration or inhibition of the translation process (60). However, There have been reports of connections to other regions, such as the 5’ UTR, coding sequence, and gene regulators (59).

Transcripts longer than 200 nucleotides are commonly referred to as long non-coding RNAs (lncRNAs), making up 80%~90% of all ncRNAs (61). In comparison to miRNA, lncRNA is larger with complex spatial structures and diverse mechanisms. It interacts with DNA and mRNA with actions including direct binding to gene promoters, mediating histone modifications and chromatin remodeling, regulating mRNA splicing, or forming endogenous siRNA. Studies have demonstrated that most long RNAs (lncRNAs) are predominantly localized in the nucleus. However, some lncRNA also exhibit functional roles in the cytoplasm (62). Additionally, certain lncRNA have the capability to be transported to neighboring cells or found in the serum through exosome-mediated transport (63). The regulatory influence of lncRNA on target gene expression is primarily achieved through cis-regulation or trans-regulation mechanisms (64). It has been estimated that there are over 60,000 lncRNA identified in humans, and this number continues to increase rapidly.

circRNAs are an RNA molecule kind found in a covalently closed, single-stranded structure that is existing in a wide variety of organisms, including viruses and mammals (65). The closed-loop structure of circRNA provides it with resistance against RNA degradation pathways. Additionally, circRNAs serve as miRNA sponges, exerting regulatory control over gene expression (66). Many circRNAs have been observed within cell nuclei, and the interactions between circRNA-miRNA-mRNA play crucial roles in multiple signaling pathways, including those associated with apoptosis, invasion, vascularization, and metastasis (67). Numerous investigations have revealed their unique patterns of expression and pivotal roles in a spectrum of conditions, encompassing, but not restricted to cancer, cardiovascular disease, neurological disorders, and autoimmune diseases (68).

### 3.3 ncRNA loading into EVs and subsequent release and uptake

The RNA composition of EVs exhibits variability contingent upon the cell type, the EV subpopulation, and the healthy or sick state of the original cells, including the stimuli they receive (69). To be moved via EVs, ncRNAs initially undergo sorting and encapsulation processes within the EVs (14). Numerous methods have been suggested for the entry of RNA into EVs, primarily categorized into two main classes: RNA-binding proteins (RBPs) and membrane proteins related to EV biogenesis.

RBPs selectively attach to particular RNA molecules to facilitate their sorting into EVs (70). Santangelo et al. (71) discovered that the RNA-binding protein Synaptotagmin-binding cytoplasmic RNA-interacting protein, or SYNCRIP, is an essential part of the sorting apparatus responsible for exocellular vesicular miRNAs in hepatocytes. The depletion of SYNCRIP was demonstrated to hinder the uptake of miRNAs into EVs. In a significant investigation, the protein heterogeneous nuclear ribonucleoprotein A2B1 (hnRNPA2B1) was identified as a regulator of both miRNAs sorting into EVs and the processing of miRNA transcripts (72). The Y-Box Binding (YBX) protein is another well-documented RNA-binding protein that facilitates the sorting of RNA into EVs. YBX-1 has been demonstrated to play a role in packaging miR-223 and miR-133. By employing a YBX-deficient cell line and thermostable group II intron reverse transcriptase sequencing (TGIRT-seq), Shurtleff et al. (73) conducted additional research, uncovering an extensive function of YBX in the categorization of not just miRNAs but also tRNAs.

Regarding membrane proteins, the ESCRT pathway is renowned for its involvement in sorting proteins into EVs (74, 75). Ago2, frequently engaged in the transport and processing of miRNAs, forms a complex that facilitates the incorporation of Alix along with Ago2-associated miRNAs into EVs (76). Wozniak et al. further validated the link between the ESCRT complex and the selective loading of RNA (77).

Following the delivery of MVBs to the cell’s plasma membrane, they adhere to a standard process of vesicular docking and fusion with the cell membrane (78). Molecular toggles (small GTPase), the cellular scaffolding (microtubule and microfilament), molecular transporters (dynein and kinesin), and the membrane fusion machinery (SNARE complex) are important elements in this mechanism (79). By overseeing every essential stage in membrane trafficking, the Rab GTPase with over 70 subtypes positioned on membrane surfaces, plays a critical role in enabling them to govern various aspects of vesicle traffic like fusion, motility, and budding (80). EVs interact with recipient cells in the extracellular space after they are released, delivering cargo to produce functional effects. Simultaneously, recipient cells take up these EVs through various mechanisms (81). Recent studies increasingly indicate that endocytosis is the predominant mechanism for the uptake of EVs (79). Cytochalasin D is a metabolite recognized for its ability to depolymerize the actin filament network, thereby inhibiting endocytic pathways (82). Treatment with Cytochalasin D has been observed on numerous occasions across different cell types, indicating a substantial reduction in EV uptake in a dose-dependent manner, although complete prevention is not achieved (83). The transmembrane ligands located on the surface of EVs also have the capability to directly attach to recipient surface receptors on cells to start signaling pathways that are downstream that activate the target cell (84). Surface molecules on EVs, including tetraspanins, immunoglobulins, proteoglycans, and lectin receptors, participate in the binding of EVs to target cells through mechanisms that remain largely unclear (85).

## 4 EV-associated ncRNA-based regulation of microglia polarization in traumatic brain injury

Traumatic brain injury is a prevalent cause of both mortality and disability, impacting individuals across all age groups (86, 87). Approximately 69 million individuals are projected to experience traumatic brain injury annually worldwide. These cases can be classified into mild (approximately 80%), moderate (approximately 10%), and severe (approximately 10%) based on clinical factors and severity (87, 88). Following traumatic brain injury, the constantly active cells typically initiate a response within minutes directed toward the damaged sites. They play a crucial role in modulating neuroinflammatory responses and secondary cascades post-injury. Activation of microglia is acknowledged as a pivotal cellular mediator in the pathophysiology of traumatic brain injury (89). Clinical studies provide evidence that activated microglia are commonly observed in the brain during the acute (90), sub-acute (91), and even chronic phases following the initial brain trauma (92). This is particularly evident after moderate to severe traumatic brain injury (92, 93). Similarly, preclinical studies have indicated that swift microglial reactivity is observed within minutes in animal models of both mild and severe traumatic brain injury (94). This process can persist for days, weeks, and even months after experimental traumatic brain injury, varying based on the types of lesions involved (95). Remarkably, an analysis of the temporal dynamics of microglia polarization following traumatic brain injury revealed the activation of both M1-like and M2-like polarized microglia during the initial stages after the injury (96). However, at one week post-injury, the M2-like phenotype transitions to the M1-like phenotype, marked by elevated levels of NOX2 expression (96). Inhibiting NOX2 in microglia, thereby altering the M1-/M2-like balance in favor of the M2-like phenotype, significantly decreased oxidative damage in the injured cortex. This demonstrates that repolarizing microglia toward an M2-like phenotype led to a reduction in oxidative damage in neurons (96).

It has been clarified that mounting ncRNAs (such as lncRNA LINC00707 (97), miR-186-5p (98), lncRNA HOXA11-AS (99), lncRNA KCNQ1OT1 (100), LncRNA Meg3 (101), lncRNA HOTAIR (102), miR-let-7c-5p (103)) are involved in modulating the microglia polarization in traumatic brain injury. The mechanisms governing microglia polarization after traumatic brain injury involve a meticulously orchestrated process regulated by various factors. These factors include but are not limited to, MYD88, miR-7a-5p, NF-κB, miR-124-3p, MDK, NLRP3, ASC, caspase-1, and miR-30a-5p. Most lncRNAs have been illustrated to regulate microglia polarization by modulating microRNA. Hu et al. (97) demonstrated that the suppression of lncRNA LINC00707 mitigates brain injury by targeting miR-30a-5p, thereby regulating microglia inflammation. Meng et al. (101) uncovered that lncRNA Meg3 intensifies microglial activation and the inflammatory response triggered by LPS and ATP stimulation. Elevating miR-7a-5p levels alleviated lncRNA Meg3-induced microglial activation without impacting lncRNA Meg3 expression. Bioinformatic analysis and dual-luciferase assays confirmed that lncRNA Meg3 directly interacts with miR-7a-5p, leading to a negative regulatory effect on miR-7a-5p expression.

**Figure 1:**
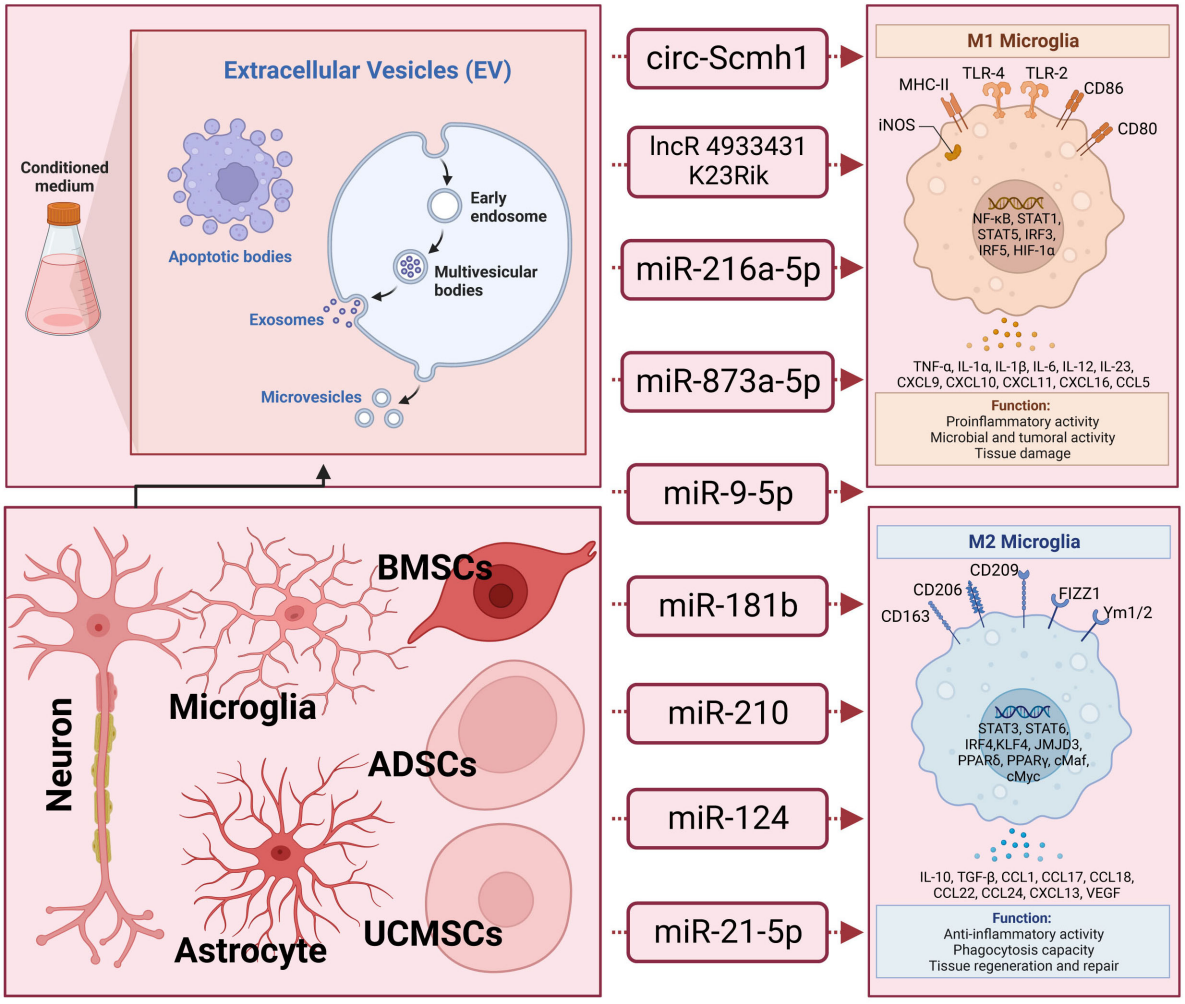
The underlying action of extracellular vesicle-noncoding RNA on the modulation of macrophage/microglia polarization in traumatic brain injury. Extracellular vesicles (EVs), encompassing exosomes, microvesicles, and apoptotic bodies, form a diverse category of lipid bilayer-encased structures released by nearly all cells. They play crucial roles in facilitating intercellular communication. These EVs carry non-coding RNAs (ncRNAs) that can either promote or inhibit the polarization of M2 microglia. Examples of such ncRNAs include circ-Scmh1, lncRNA 4933431K23Rik, miR-216a-5p, miR-873a-5p, miR-9-5p, miR-181b, miR-210, miR−124, and miR-21-5p. These ncRNA-containing EVs have been identified across a spectrum of cell types, including neurons, astrocytes, microglia, and stem cells derived from various tissues such as bone marrow and adipose tissue.

Moreover, it has been elucidated that EVs derived from different stem cells, including human adipose mesenchymal stem cells (104), human umbilical cord mesenchymal stem cells (105), and stem cells from human exfoliated deciduous teeth (106), play a role in modulating microglia polarization in traumatic brain injury. In discussions about EV-derived ncRNAs, a growing body of literature has emphasized their significance in regulating microglia polarization. EVs containing ncRNAs that either promote or inhibit M2 microglia polarization, such as circ-Scmh1 (107), lncRNA 4933431K23Rik (108), miR-216a-5p (109), miR-873a-5p (110), miR-9-5p (111), miR-181b (112), miR-210 (113), miR−124 (10), and miR-21-5p (114), have been identified from diverse cell sources including neurons, astrocytes, microglia, and stem cells differentiated from various tissues like bone marrow and adipose tissue. Notably, a significant proportion of these EV-ncRNAs are derived from stem cells. EV-miR-216a-5p (109), miR-181b (112), miR-210 (113), and miR−124 (10) derived from mesenchymal stem cells are involved in repairing traumatic injury by inducing microglial M2 polarization. EVs derived from adipose-derived stem cells have been shown to improve nerve damage in the hippocampus resulting from post-traumatic brain injury. This therapeutic effect is attributed to the delivery of circ-Scmh1, which promotes microglial M2 polarization (107). Indeed, not all EV-associated ncRNAs necessarily facilitate the polarization of M2-type microglial cells. Based on research by Yin and his colleagues (114), microglia phagocytosed neuron-derived EVs containing miR-21-5p, leading to the induction of microglial polarization. Simultaneously, the expression of miR-21-5p increased in M1 microglial cells. The polarization of M1 microglia exacerbated the release of neuroinflammatory factors, hindered neurite outgrowth, elevated the accumulation of P-tau, and induced apoptosis in neurons. In-depth information pertaining to this section can be referenced in **Figure 1** and **Table 1**.

**Table 1:** Preclinical studies assessing the effects of ncRNAs transferred via EVs in models of traumatic brain injury.

Author, [Ref.]	ncRNA	Expression	Purification	Donor cell	Injury model	Effect on microglia activation
**Chen et al., 2023 (** 107)	circ-Scmh1	Upregulation	Centrifuge Filter Unit	Adipose-stem cell	Controlled cortical impact	Ameliorate nerve damage and promote microglial M2 polarization
**He et al., 2023 (** 108)	lncRNA 4933431K23Rik	Upregulation	Kit	Astrocyte	Controlled cortical impact	Improve Post-traumatic Recovery and microglial M2 polarization via SMAD7
**Liu et al., 2020 (** 109)	miR-216a-5p	Upregulation	Centrifuge Filter Unit	Mesenchymal stem cells	Controlled cortical impact	Repair traumatic injury by shifting microglial M1/M2 polarization
**Long et al., 2020 (** 110)	miR-873a-5p	Upregulation	Ultracentrifugation	Astrocyte	Controlled cortical impact	Inhibit neuroinflammation via microglia phenotype modulation
**Wang et al., 2023 (** 111)	miR-9-5p	Upregulation	Ultracentrifugation	Microglia	Lipopolysaccharide	Reduce LPS-stimulated inflammation in microglia cells
**Wen et al., 2022 (** 112)	miR-181b	Upregulation	Ultracentrifugation	Mesenchymal stem cells	Fluid percussion injury	Reduce apoptosis and inflammation and promote microglial M2 polarization
**Xiong et al., 2023 (** 113)	miR-210	Upregulation	Ultracentrifugation	Mesenchymal stem cells	Lipopolysaccharide	Inhibit neuronal inflammation and contribute to neurite outgrowth through modulating microglia polarization
**Yang et al., 2018 (** 10)	miR−124	Upregulation	kit	Mesenchymal stem cells	Controlled cortical impact	Promote the M2 Polarization and enhance hippocampus neurogenesis via TLR4 pathway
**Yin et al., 2020 (** 114)	miR-21-5p	Upregulation	Ultracentrifugation	Neuron	Controlled cortical impact	Promote polarization of M1 microglia in culture

ncRNA, Non-coding RNAs; LncRNA, long non-coding RNAs; TLR4, Toll-like receptors 4.

## 5 EV-associated ncRNA-based regulation on macrophage polarization in lung injury

Acute lung injury is a clinical syndrome marked by damage to alveolar epithelial and lung capillary endothelial cells, heightened alveolar-capillary permeability, and impaired gas exchange (115). Despite considerable scientific and technological progress, lung injury continues to present a significant threat to human life and health. Its rapid clinical development, high pathogenic rate, limited effective treatment options, poor prognosis, and elevated mortality rate highlight the urgent need for research dedicated to identifying potential clinical solutions aimed at improving and potentially eradicating lung injury (115). Recently, a growing body of evidence suggests that macrophages play a pivotal role in the pathogenesis of lung injury. Macrophages can be categorized into two types: M1, associated with classical activation, and M2, associated with alternative activation (115). It has been elucidated that various ncRNAs (such as miR-223, miR-142, miR-146a, miR-155) participate in modulating macrophage polarization in the context of lung injury. The down-regulation of miR-223/142 has been observed, and its overexpression has been shown to inhibit macrophage activation, lung inflammation, and apoptosis (116, 117). miRNA-146a plays a regulatory role in the M2-type polarization of macrophages and immune response control by targeting Notch1, IRAK1, and IRF5, thereby enhancing the anti-inflammatory effect (118–120). miRNA-155 is upregulated in the LPS-induced acute lung injury model, and inhibiting its expression has been shown to have anti-inflammatory effects by suppressing the release of pro-inflammatory factors (121). Additionally, it has been observed that miR-155 promotes M1 macrophage activation, thereby exacerbating lung inflammation and tissue damage in macrophages through the down-regulation of C/EBPβ (115, 122).

**Figure 2:**
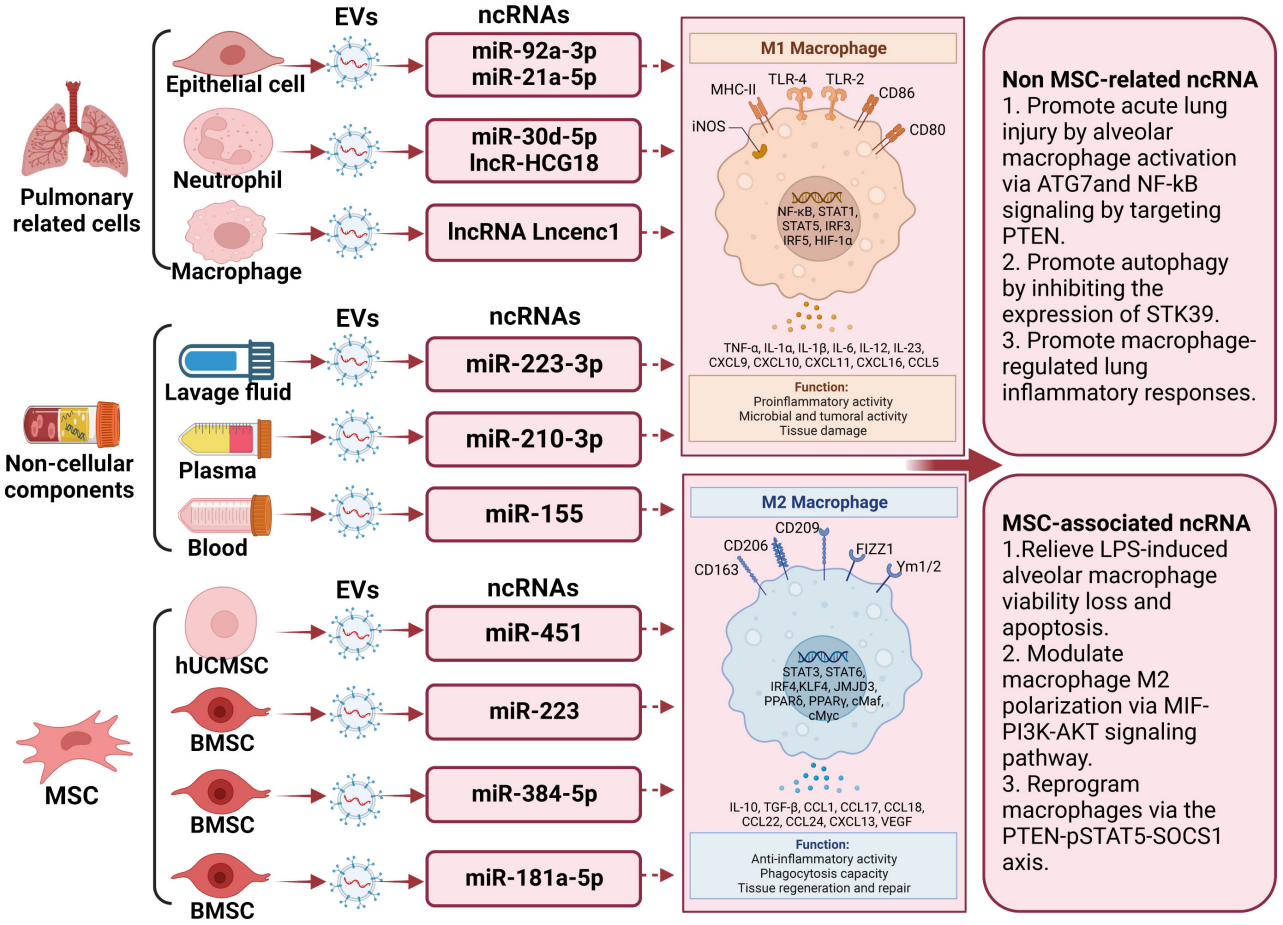
The underlying action of extracellular vesicle-noncoding RNA on the modulation of macrophage/microglia polarization in lung injury. Extracellular vesicles (EVs) harboring non-coding RNAs (ncRNAs) capable of either promoting or inhibiting M2 microglia polarization have been recognized. Examples of these ncRNAs include lncRNA Lncenc1, miR-223-3p, miR-155, miR-30d-5p, miR-221, miR-320a, miR-210-3p, miR-384-5p, miR−92a-3p, miR-451, miR-181a-5p, miR-21a 5p, miR‐223, and LncRNA HCG18. These EVs have been isolated from diverse cellular origins, including macrophages, bronchoalveolar lavage fluid, mouse blood, epithelial cells, human plasma, polymorphonuclear neutrophils, and stem cells derived from tissues such as bone marrow and adipose tissue. Among these non-coding RNAs, those released by stem cells can promote the differentiation of M2 macrophages, thereby improving the prognosis of lung injury.

In discussions about EV-derived ncRNAs, a growing body of literature has emphasized their significance in regulating microglia polarization. EVs containing ncRNAs that either promote or inhibit M2 microglia polarization, such as lncRNA Lncenc1 (123), miR-223-3p (116), miR-155 (122), miR-30d-5p (124), miR-221 (125), miR-320a (125), miR-210-3p (126), miR-384-5p (127), miR−92a-3p (128), miR-451 (9), miR-181a-5p (129), miR-21a-5p (130), miR‐223 (8), and LncRNA HCG18 (131), have been identified from diverse cell sources including macrophage, bronchoalveolar lavage fluid, mouse blood, epithelial cells, human plasma, epithelial, polymorphonuclear neutrophil, and stem cells differentiated from various tissues like bone marrow and adipose tissue. The regulation of macrophage polarization following lung injury is a precisely coordinated process influenced by a range of factors. These factors encompass, among others, Notch2/SOCS1, PTEN, pSTAT5, MIF, PI3K, AKT, and ATG7. Significantly, a substantial portion of these EV-derived ncRNAs originates from mesenchymal stromal cells. miR-384-5p (127), miR-451 (9), miR-181a-5p (129), and miR-223 (8), carried by mesenchymal stromal cells, participate in the repair of traumatic injuries by promoting microglial M2 polarization. Of note, miR-221 (125), miR-320a (85), and miR−92a-3p (128) transported by EVs originating from epithelial cells stimulate alveolar macrophage activation. Concurrently, miR-21a-5p (130), transported by EVs derived from epithelial cells, promotes M2 macrophage polarization through the Notch2/SOCS1 pathway. In addition, EV-associated lncRNA Lncenc1from macrophage, EV-associated miR-223-3p from bronchoalveolar lavage fluid (116), EV-associated miR-155 from mouse blood (122), EV-associated LncRNA HCG18 and miR-30d-5p from polymorphonuclear neutrophils (124, 131), and EV-associated miR-210-3p (126) from human plasma aggravate acute lung injury by promoting M1 macrophage polarization. Comprehensive details regarding this section can be consulted in **Figure 2** and **Table 2**.

**Table 2:** Preclinical studies assessing the effects of ncRNAs transferred via EVs in models of lung injury.

Author, [Ref.]	ncRNA	Expression	Purification	Donor cell	Injury model	Effect on macrophage activation
**Han et al., 2023 (** 123)	lncRNA Lncenc1	Upregulation	Ultracentrifugation	Macrophage	LPS-induced lung injury	Promote inflammasome activation in macrophage
**He et al., 2022 (** 116)	miR-223-3p	Upregulation	Ultracentrifugation	Bronchoalveolar lavage fluid	LPS-induced lung injury	Promote autophagy by inhibiting the expression of STK39
**Jiang et al., 2019 (** 122)	miR-155	Upregulation	Ultracentrifugation	Mouse blood	Sepsis-related ALI	Promote proliferation and inflammation by targeting SHIP1 and SOCS1
**Jiao et al., 2021 (** 124)	miR-30d-5p	Upregulation	Kit	Polymorphonuclear neutrophils	Sepsis-related ALI	Promote M1 macrophage activation and macrophage pyroptosis via NF-κB
**Lee et al., 2016 (** 125)	miR-221 miR-320a	Upregulation	Ultracentrifugation	Epithelial cells	Hyperoxia-induced oxidative stress	Promote macrophage-regulated lung inflammatory responses
**Li et al., 2021 (** 126)	miR-210-3p	Upregulation	Ultracentrifugation	Human plasma	Sepsis-related ALI	Enhance macrophage inflammation and apoptosis via ATG7
**Liu et al., 2021 (** 127)	miR-384-5p	Upregulation	Ultracentrifugation	Mesenchymal stromal cells	LPS-induced lung injury	Relieve LPS-induced alveolar macrophage viability loss and apoptosis
**Liu et al., 2021 (** 128)	miR−92a-3p	Upregulation	Ultracentrifugation	Epithelial cells	Sepsis-related ALI	Promote alveolar macrophage activation via NF-kB signaling by targeting PTEN
**Liu et al., 2022 (** 9)	miR-451	Upregulation	Kit	Mesenchymal stromal cells	Severe burn rat model	Modulate macrophage M2 polarization via MIF- PI3K-AKT signaling pathway
**Su et al., 2022 (** 129)	miR-181a-5p	Downregulation	Ultracentrifugation	Mesenchymal stromal cells	Lipopolysaccharide	Reprogram macrophages via the PTEN-pSTAT5-SOCS1 axis
**Wang et al., 2022 (** 130)	miR-21a-5p	Upregulation	Ultracentrifugation	Epithelial cells	Mechanical ventilation	Promote M2 macrophage polarization via Notch2/SOCS1
**Xu et al., 2023 (** 8)	miR‐223	Upregulation	Ultracentrifugation	Mesenchymal stem cells	Lipopolysaccharide	Ameliorate acute lung injury by regulating macrophage M2 polarization
**Zhu et al., 2023 (** 131)	LncRNA HCG18	Upregulation	Ultracentrifugation	Polymorphonuclear neutrophil	Sepsis acute lung injury	Aggravate sepsis acute lung injury by regulating macrophage polarization

ncRNA, Non-coding RNAs; LncRNA, long non-coding RNAs; ALI, acute lung injury.

## 6 EV-associated ncRNAs involved in both conditions demonstrate significant overlaps in their modes of action

Despite the diverse pathophysiological processes occurring in different tissues and organs associated with these two disorders, a common feature is the role of EVs as carriers for regulating macrophage/microglia polarization via ncRNA mechanisms. Interestingly, certain EV-associated ncRNAs seem to participate in both conditions by modulating respective signaling pathways. These overlapping ncRNAs demonstrate different protective effects on disease outcomes. Giving significant attention to uncovering potential shared ncRNAs may offer new insights into tissue remodeling processes and help identify therapeutic targets for lung injury and traumatic brain injury. Based on the aforementioned intervention studies involving EV-associated ncRNAs, a total of 2 ncRNAs (miR-210 and miR-21) have also been identified, with strong evidence suggesting their involvement in both pathophysiological conditions. miR-210 is considered the primary hypoxia-inducible microRNA, as it has been consistently observed to exhibit significant upregulation in response to hypoxic conditions across various cell types (132). In addition to its role in the intricate regulation of various biological processes, miR-210 loaded into EVs has also been implicated in the treatment of several human diseases, including acute lung injury and traumatic brain injury. Based on the study conducted by Xiong et al. (113), in the condition of traumatic brain injury, EVs originating from mesenchymal stem cells, which are engineered to overexpress miR-210, exhibit the ability to suppress neuronal inflammation and promote neurite outgrowth by modulating microglia polarization. On the contrary, EVs derived from plasma deliver miR-210-3p, targeting ATG7, to promote sepsis-induced acute lung injury by regulating autophagy and activating inflammation (126). With regard to the miR-21, one of the most extensively studied in humans, it plays crucial roles in various physiological and pathological processes, including cell proliferation, apoptosis, cell migration and invasion, and gene expression modulation. miR-21 is often found to be highly expressed in many cell types and is considered a potential driver of injury development and progression. EVs derived from neurons, featuring elevated levels of miR-21-5p, facilitated the polarization of M1 microglia in cultured conditions (114). On the contrary, EVs originating from epithelial cells, featuring elevated levels of miR-21-5p, foster M2 macrophage polarization through the Notch2/SOCS1 pathway during mechanical ventilation (130). This suggests that the therapeutic effects of a particular ncRNA may differ depending on the cells from which it is secreted or the type of disease it is involved in.

## 7 EV-associated ncRNAs represent promising novel candidates for both therapeutic objectives and diagnostics

While there have been notable strides in comprehending the sequence of events that dictate the pathophysiology of lung and traumatic brain injuries, the fundamental mechanisms still await complete clarification. Currently, there are no biological tools available for the detection of lung and traumatic brain injuries or for monitoring tissue recovery. The urgent requirement for innovative diagnostics in identifying lung and traumatic brain injuries and predicting deterioration risk in such injury patients has led to the exploration of endogenous markers. Numerous EV-ncRNAs exhibit abnormal expression following injury, suggesting their potential as biomarkers. Elevated or diminished levels of EV-ncRNA detected in lung and traumatic brain injuries play a role in the diagnostic process. In the context of traumatic brain injury, as indicated by Harrison et al. (133), there is an upregulation of EV-associated miR-21, miR-146, miR-7a, and miR-7b, derived from traumatic brain injury mice, while miR-212 experiences a downregulation (133). Furthermore, some researchers illustrated that EVs originating from the brain and containing ncRNA could be employed to characterize distinct states of traumatic brain injury and discern potential signaling pathways in the human brain following damage (134). Crucially, they proposed the potential utilization of a biomarker panel to delineate the characteristics of a particular lesion, instead of relying on just one marker. This approach acknowledges that a solitary marker might lack the ability to differentiate the intricate stages of a lesion and sufficient particularity for healing (134). EV-ncRNA derived from plasma not only serves as a diagnostic indicator for traumatic brain injury but also mirrors the underlying pathophysiological processes following traumatic brain injury. The pattern of miRNA activity in EVs derived from peripheral blood plasma was investigated in an inducement of traumatic brain damage in rats using high-throughput whole transcriptome sequencing, followed by subsequent bioinformatics analysis. Consequently, Wang et al. (135) detected 50 miRNAs with significant differential expression, comprisingmiRNAs with 31 increased level and 19 decreased level. Similarly, the researchers showcased that certain EV-associated ncRNAs originating from bodily fluids can serve as early diagnostic indicators for acute lung injury to a certain extent. Parzibut et al. (136) conducted a study in which they examined the plasma EV-miRNA expression in eight patients with acute respiratory distress syndrome and ten healthy subjects. Significantly, among these miRNAs, 12 were identified as having differential expression, and seven (miR-221-3p, miR-24-3p, miR-130a-3p, Let-7d-3p, miR-1273a, miR-98-3p, and miR-193a-5p) were proven to effectively distinguish between acute respiratory distress syndrome and hemorrhagic shock. Furthermore, within the cohort of patients with acute type A aortic dissection, plasm EVs from individuals with acute lung injury showed upregulation of miR-485 and downregulation of miR-206 compared to those without acute lung injury. This observation suggests the potential of miR-485 and miR-206 to serve as a marker for acute lung damage in individuals suffering from acute An aortic dissection (137).

EV-associated ncRNAs serve not only as biomarkers for diagnosing lung and traumatic brain injuries but also hold promise as potential therapeutic targets. The employment of carrier systems to deliver therapeutic payloads to specific cells or tissues has garnered significant interest. Due to their capability to transport cargo, EVs have emerged as prominent entities in the field of nanotherapeutics. Modifying ncRNAs with expression from EVs and delivering them to recipient cells and tissues could offer insightful information about the emergence of lung and traumatic brain injuries, rendering them appealing targets for therapeutic intervention. As indicated by Long et al. (110), EVs derived from brain extracts-stimulated astrocytes were found to promote the transformation of microglial M2 phenotype early on in the course of traumatic brain injury. Within these astrocyte-derived EVs, more than 100 miRNAs were identified. Notably, miR-873a-5p emerged as a significant component, exhibiting high expression levels in human traumatic brain tissue. Additionally, some studies have suggested that ncRNA in EVs can promote the microglial cells’ transition to the M2 subtype, thereby facilitating the neuronal function returning following a catastrophic brain injury. miR-124 stands out as a miRNA unique to the brain with high expression in microglia (138). In a state of normal physiological function, miR-124 governs the functionality of microglia and holds a pivotal role in maintaining their quiescent state (139). Under pathological conditions, the decrease in miR-124 expression amplifies inflammation of the brain caused by inducing microglial polarization toward the M1 phenotype. Conversely, the elevation of miR-124 expression diminishes neuroinflammation by encouraging polarization of microglia into the M2 subtype (140, 141). In line with prior investigations into the role of EV- miR-124 in brain recovery following traumatic brain injury, recent research by Yang et al. (10) has shown that EVs can act as an efficient delivery tool for miR-124 to the brain. This delivery contributes to the stimulation of microglial M2 polarization, leading to enhanced, following brain damage, enhanced functional recovery and hippocampus neurogenesis. This is attributed to their nano-size advantages, capability to transport microRNA, and ability to traverse the blood-brain barrier. For lung injury, stem cells carrying ncRNA hold tremendous therapeutic potential. For example, Liu et al. (9) indicated that EV-miR-451 derived from human umbilical cord mesenchymal stem cells mitigated acute lung injury by influencing macrophage M2 polarization through the regulation of the MIF-PI3K-AKT signaling pathway. Xu et al. (8) shown that EVs generated from bone marrow mesenchymal stem cells reduce acute lung damage caused by LPS by modulating the M2 polarization of alveolar macrophages. The validation using miR-223 mimics and inhibitors confirms the crucial role of miR-223 in the polarization of M2 macrophages mediated by EVs derived from bone marrow mesenchymal stem cells (142).

## 8 Conclusion and perspectives of the significance of EV-associated ncRNAs

EVs play a pivotal role in the progression of lung and traumatic brain injuries, largely influenced by their cargo contents. The inherent advantages of EVs have positioned them as crucial entities for utilizing EV-derived ncRNAs as diagnostic, prognostic, and therapeutic tools. Abundance in various body fluids (such as blood, urine, saliva) renders EVs easily extractable and analyzable, offering noninvasive advantages. EVs, serving as natural carriers for ncRNAs, shield these cargos from degradation by endogenous RNases. Specific biophysical characteristics, including ease of retrieval and preservation, make EVs suitable for *in vitro* studies and engineering purposes. In this context, ncRNAs encapsulated within EVs have been shown to exert biological functions by modulating specific aspects, particularly macrophage/microglia polarization. Mesenchymal stem cells represent a group of pluripotent cells with the ability for multifaceted development and self-renewal. EVs derived from Mesenchymal stem cells of various sources demonstrate promotive effects on M2 macrophage/microglia polarization through ncRNAs in both injury conditions, indicating their potential application in clinical cell-free therapy. Furthermore, EV-ncRNAs hold significant promise as medicinal or diagnostic tools, representing an exciting advancement in the management of lung and traumatic brain injuries. However, considerable challenges must be addressed before EV-ncRNAs can be implemented as clinical assays in such disorders. The mechanism of action of EV-ncRNAs is primarily grounded in animal and cellular models, necessitating further validation in medical specimens. Prospective studies based on medical samples often rely on small cohorts and lack validation in larger cohort studies. Further exploration is necessary to develop a technique capable of generating pure and homogeneous EVs. It is crucial to acknowledge that different isolation protocols may result in the extraction of subpopulations of EVs with varying ncRNAs (143–145). In summary, while EV-ncRNAs show promise as valuable biomarkers and therapeutic agents for lung and traumatic brain injuries, continued research efforts and clinical validation are essential to fully harness their potential in clinical practice.

## Author contributions

ZC: Conceptualization, Data curation, Formal Analysis, Investigation, Methodology, Project administration, Resources, Software, Writing – original draft, Writing – review & editing. JZ: Data curation, Investigation, Methodology, Resources, Software, Supervision, Writing – original draft. YP: Data curation, Investigation, Methodology, Software, Writing – original draft. ZH: Data curation, Investigation, Project administration, Software, Writing – original draft. SL: Conceptualization, Data curation, Funding acquisition, Investigation, Methodology, Project administration, Resources, Software, Supervision, Validation, Visualization, Writing – original draft, Writing – review & editing.
